# Editorial: Molecular aspects of natural products in diabetic complications

**DOI:** 10.3389/fendo.2024.1431775

**Published:** 2024-07-04

**Authors:** Ankit P. Laddha, Manisha J. Oza, Yogesh A. Kulkarni

**Affiliations:** ^1^ Department of Pharmaceutical Sciences, University of Connecticut, Storrs, CT, United States; ^2^ Lemole Center for Integrated Lymphatics and Vascular Research, Lewis Katz School of Medicine, Temple University, Philadelphia, PA, United States; ^3^ Shobhaben Pratapbhai Patel School of Pharmacy & Technology Management, SVKM‘s NMIMS, Mumbai, India

**Keywords:** natural products, diabetic complications, diabetes, flavonoids, inflammation

Diabetes mellitus, characterized by elevated blood glucose levels over extended periods, presents a persistent challenge in modern healthcare (1). According to the recent International Diabetic Federation (IDF) Global Atlas, a continued global increase in diabetes prevalence confirms diabetes as a significant global challenge to the health and well-being of individuals, families, and societies. There are currently 537 million adults (20–79 years) living with diabetes, and it has been estimated that the number will reach 643 million by 2030 and 783 million by 2045 (2). The pathophysiology of diabetes revolves around three pivotal conditions: poor metabolic control, inadequate insulin action and insulin production by pancreatic beta cells, and prolonged elevation of blood glucose levels (3).

The physiological consequences of prolonged hyperglycemia are diverse, ranging from microvascular impairments like blindness, nephropathy, and neuropathy to macrovascular complications such as cardiovascular diseases and stroke (4). The prevalence of chronic complications significantly increases with age and duration of diagnosed diabetes.

Non-enzymatic glycation, initiated by the interaction between reducing sugars and proteins, leads to the formation of advanced glycation end-products (AGEs). These compounds, formed through a series of reactions, contribute to tissue damage and functional impairments characteristic of long-term diabetes. Pathogenesis of diabetic complications involves complex biochemical processes at the cellular level with the involvement of several signaling pathways. Increased oxidative stress in various types of cells, activation of specific enzyme pathways like the polyol and hexosamine pathway, and dysregulation of protein kinase C are pivotal in mediating tissue damage and dysfunction. Moreover, a network of inflammatory mediators and growth factors, including NF-kβ, TGF-β, IL-6, and VEGF, plays an important role in the progression of diabetic complications. These molecules modulate intracellular signaling cascades, exacerbating tissue damage and promoting the development of pathologies such as nephropathy, retinopathy, neuropathy, and cardiomyopathy (5). These complications underscore the urgent need for effective management and prevention strategies. In recent decades, there have been notable advancements in the treatment of diabetes mellitus and its complications. The treatment of diabetic complications typically involves a combination of medical interventions, lifestyle modifications, and ongoing management to prevent further progression and improve quality of life. Management of diabetic neuropathy often includes medications such as anticonvulsants, antidepressants, or opioids to control pain, whereas laser therapy, intraocular injections, and anti-VEGF drugs are commonly used for the treatment of retinopathy. Angiotensin-converting enzyme (ACE) inhibitors or angiotensin receptor blockers (ARBs) are commonly prescribed to protect kidney function. However, conventional anti-diabetic medications are associated with various adverse events that affect liver and kidney function. Recognizing the limitations of conventional treatments, the World Health Organization (WHO) mentioned the importance of medicinal plants in dietary regimens for managing diabetes mellitus. Many medicinal plants are being used in various countries in various forms for the management of diabetes. Natural product research has emerged as a promising avenue for combating various diseases, including diabetes and its complications, due to the reduced toxicity and minimal side effects. Different types of secondary metabolites such as alkaloids, glycosides, tannins, and polyphenols have been systematically studied for their possible effects on diabetes and diabetic complications. Preclinical studies are the main focus of research on the role of natural products in diabetes and its complications. Many researchers are now exploring combinations of synthetic molecules and natural products to mitigate synthetic drug toxicity (6). Nonetheless, there remains a significant knowledge gap regarding the molecular and cellular mechanisms underlying the therapeutic effects of natural products in conditions like diabetes and its complications. Also, systematic clinical research is needed with respect to natural products for diabetes and diabetic complications.

Through the current Research Topic, we wanted to address the knowledge gap in understanding the molecular mechanism of diabetes and diabetic complications and the role of natural products against their progression.

This current Research Topic comprises four specific contributions. An article by Zhu et al. presents Mendelian randomization analysis which elucidated a direct causal relationship between type 2 DM (T2DM) and male infertility (MI) as well as erectile dysfunction (ED), emphasizing the genetic mechanisms underlying T2DM-related complication. A contribution by Daher et al. presents a longitudinal cohort study in individuals with type 1 DM (T1D), revealing a significant association between a genetic variant in the melatonin receptor 1A gene and renal function decline, highlighting a novel link between the melatonin system and microvascular complications in diabetes. A systematic review and meta-analysis by Zou et al. focused on the effects of *Ginkgo biloba* on T2DM patients, indicating potential benefits for peripheral arterial and cardiovascular health. Lastly, research by Tian et al. highlighted promising effects of hirudin, a substance derived from leeches, in managing diabetic nephropathy through its anti-coagulant, anti-fibrotic, and anti-inflammatory properties. These findings collectively contribute to our understanding of DM pathogenesis and offer insights into potential therapeutic approaches for its complications.

## Author contributions

AL: Writing – review & editing, Writing – original draft. MO: Writing – review & editing, Writing – original draft. YK: Writing – review & editing, Writing – original draft.
